# Hemodiafiltration beyond the CONVINCE trial

**DOI:** 10.1093/ckj/sfag115

**Published:** 2026-04-15

**Authors:** Giovanni F M Strippoli, Krister Cromm, Vasileios C Pezoulas, Nikolaos Tachos, Dimitrios I Fotiadis, Allison Jaure, Jolanta Malyszko, Michael Anger, Felix Fischer, Jörgen Hegbrant, Sagar Nigwekar, Rümeyza Kazancıoğlu

**Affiliations:** Sydney School of Public Health, The University of Sydney, NSW, Australia; Department of Precision and Regenerative Medicine and Ionian Area (DIMEPRE-J), University of Bari Aldo Moro, Bari, Italy; Center for Patient-Centered Outcomes Research, Department of Psychosomatic Medicine, Charité – Universitätsmedizin Berlin, corporate member of Freie Universität Berlin and Humboldt-Universität zu Berlin, Berlin, Germany; Fresenius Medical Care Deutschland GmbH, Global Medical Office, Bad Homburg, Germany; Unit of Medical Technology and Intelligent Information Systems, Department of Materials Science and Engineering, University of Ioannina, Ioannina, Greece; Biomedical Research Institute - FORTH, Ioannina, Greece; Unit of Medical Technology and Intelligent Information Systems, Department of Materials Science and Engineering, University of Ioannina, Ioannina, Greece; Biomedical Research Institute - FORTH, Ioannina, Greece; Unit of Medical Technology and Intelligent Information Systems, Department of Materials Science and Engineering, University of Ioannina, Ioannina, Greece; Biomedical Research Institute - FORTH, Ioannina, Greece; Sydney School of Public Health, The University of Sydney, NSW, Australia; Department of Nephrology, Dialysis and Internal Medicine, Medical University of Warsaw, Warsaw, Poland; University of Colorado Anschutz School of Medicine, Aurora, CO, USA; Fresenius Medical Care, Global Medical Office, Waltham, MA, USA; Center for Patient-Centered Outcomes Research, Department of Psychosomatic Medicine, Charité – Universitätsmedizin Berlin, corporate member of Freie Universität Berlin and Humboldt-Universität zu Berlin, Berlin, Germany; Division of Nephrology, Department of Clinical Sciences, Lund University, Lund, Sweden; Division of Nephrology, Department of Medicine, Massachusetts General Brigham, Boston, MA, USA; Harvard Medical School, Boston, MA, USA; Division of Nephrology, School of Medicine, Bezmialem Vakif University, Istanbul, Türkiye

**Keywords:** convection, digital-twin, guideline, hemodiafiltration, review

## Abstract

Online hemodiafiltration (HDF) is a dialysis modality that can improve patient outcomes beyond those achieved with conventional high-flux hemodialysis (HD) in patients with kidney failure. The CONVINCE trial, the latest and the largest of a series of randomized trials comparing HDF with HD, demonstrated a 23% reduction in the risk of all-cause mortality with high-volume HDF compared to high-flux HD. Systematic reviews of the totality of evidence confirmed cardiovascular and survival benefits of HDF. Further hard-endpoint trials are unlikely to change these findings. The challenge is implementation of these findings in clinical care. Nephrology is used to timeframes of 10–15 years before innovation gets implemented at the bedside, and this may well apply to HDF, particularly in regions where structural, regulatory, and financial barriers persist. In this narrative review, we discuss in broad terms the key items of a forward-looking research agenda for HDF after CONVINCE. Key priorities include real-world implementation studies; a strong focus on patient-reported outcomes; mechanistic research to understand why HDF is superior to HD and exploring personalized HDF regimens. Attaining these priorities will require leveraging innovative methods and we discuss two, target trial emulation methods and use of digital twin technologies. We aim to stimulate reflection and enthusiasm toward a modern approach to dialysis research that embraces innovation and centers on patient priorities. We advocate for world-class clinical guidelines on HDF, avoidance of opinion and position statements and a decisive, creative, and innovative research path. Patients with kidney failure deserve care informed by the best available evidence, implemented through rigorous guideline processes and adapted to patient preferences, health-system context, and feasibility.

## INTRODUCTION

Despite decades of widespread use, hemodialysis (HD) has seen few breakthroughs that have translated into clear survival advantages for patients [1, 2]. Most technical and procedural refinements have not caused significant gains in patient-relevant outcomes. Hemodiafiltration (HDF) combines the diffusive clearance of standard HD with convective solute removal that enhances middle- and large-molecular-weight uremic toxins clearance. Early randomized trials and cohort studies suggested the superiority of HDF versus HD but were underpowered and had other methodological limitations [3–9]. Further rigorous, well-powered trials were demanded, and the European Union Horizon 2020 Program funded the international, multi-center, prospective, randomized, controlled study comparing high-dose HDF versus conventional high flux HD (CONVINCE, Dutch Trial Register number, NTR7138, grant no. 754803-2). This pragmatic trial enrolled 1360 participants across 61 European centers and found a 23% reduction in the risk of all-cause mortality with HDF versus high-flux HD (hazard ratio 0.77; 95% confidence interval, 0.65–0.93) [10]. Findings were consistent with previous meta-analyses, especially for reduction in cardiovascular mortality but contributed precision and external validation making the data stable and unlikely to change with additional cardiovascular mortality-powered trials (Table 1) [11–17].

**Table 1:** Empirical assessment of evidence robustness for hemodiafiltration survival advantage and requirements to nullify or reverse current findings.

Outcome	Current hazard ratio (95% confidence intervals)	Evidence stability	To nullify, showing no significant effect of hemodiafiltration versus hemodialysis (hazard ratio ≥ 1.0)	To reverse, showing harm of hemodiafiltration versus hemodialysis (hazard ratio > 1.0, *P* < .05)	Plausibility of nullification (non-significant effect)b	Plausibility of reversal (harm)b
All-cause mortality	0.84 (0.74–0.95)	Suggestive since year 2011, significant only after CONVINCE^a^, <30% heterogeneity	≥1 large RCT (*n* > 4500) with hazard ratio 1.2#	2–3 large RCTs (*n* > 10 000) with hazard ratio > 1.1	Highly unlikely	Extremely unlikely
Cardiovascular mortality	0.78 (0.64–0.96)	Stable since year 2011, 0% heterogeneity	≥1 large RCT (*n* = 3000) with hazard ratio 1.3^a^	≥2 RCTs with hazard ratio > 1.2	Highly unlikely	Extremely unlikely

Risk estimates in this table are derived from a contemporary meta-analysis of randomized controlled trials (RCTs) based on Cochrane methodology. Calculations for nullification or reversal are based on quantitative synthesis principles that incorporate trial sequential analysis and influence modeling. Specifically, we estimated the magnitude and number of additional RCTs, assuming conventional sample sizes and plausible hazard ratios, that would be required to shift the pooled hazard ratio to the null (HR ≥ 1.0) or to a statistically significant reversal indicative of harm (HR > 1.0 with *P* < .05). The projections consider meta-analytic effects, the precision of current confidence intervals, and the weight of existing evidence. The plausibility assessments integrate assumptions about feasible effect sizes in future studies, the quality of existing evidence, and clinical equipoise. Analyses are with fixed-effects model given heterogeneity = 0% for cardiovascular mortality and <30% for all-cause mortality.

^a^The international, multi-center, prospective, randomized, controlled study comparing high-dose Haemodiafiltration (HDF) versus conventional high flux Haemodialysis (HD)-CONVINCE#approximated to the closest decimal.

^b^Qualitative likelihood descriptors are used on a single ordinal scale (somewhat unlikely, unlikely, highly unlikely, extremely unlikely) to reflect relative probability that future randomized evidence would materially alter current conclusions.

In this review, we analyze what will be needed beyond CONVINCE. We outline a forward-looking research agenda to address key remaining questions.

### State of the art after CONVINCE: the totality of evidence and the need for guidelines

Several randomized controlled trials (RCTs) of HDF have not been individually well powered for the outcome of all-cause mortality, but cumulatively indicated clear benefit for cardiovascular death.

While future studies may inform subgroups or health economics, research should shift toward implementation, mechanisms, patient-reported outcomes (PROs), and personalization of HDF. Delayed translation of evidence in nephrology, as seen in anemia management despite RCT and meta-analytic harm signals [18, 19], underscores the need to prioritize the totality of evidence over isolated trials. By totality we refer to a structured and critical synthesis of available data that considers study design, risk of bias, consistency of effects across trials, precision of estimates, biological plausibility, and external validity, which is typically done by guideline agencies, rather than simple aggregation of trial findings [20].

A standard path when new evidence arises and guidelines are not promptly made available is that opinion and consensus statements are released by multiple agencies, like a recent one from the European Dialysis Working Group (EuDIAL) of the European Renal Association [21].

While the consensus statement emphasized fragility metrics (fragility index-FI, fragility quotient-FQ, survival-inferred fragility index-SIFI) to assess individual trials, such measures do not replace guideline-level evidence synthesis, which incorporates consistency, effect size, plausibility, and external validity through frameworks such as GRADE [20].

Evidence for a reduced risk of all-cause mortality with HDF is of moderate certainty when downgraded once for indirectness (i.e. limited applicability due to clinical heterogeneity in patient populations, interventions, and trial characteristics across randomized treatment arms), and would be considered of low certainty if further downgraded for imprecision and/or risk of bias. In contrast, the evidence for a reduction in cardiovascular mortality with HDF is of high certainty, or moderate certainty at worst, based on consistent findings across randomized trials and meta-analyses (Table 2) [11, 13, 14].

**Table 2:** Summary of findings table for the effects of hemodiafiltration versus hemodialysis on all-cause and cardiovascular mortality based on contemporary reviews of the evidence [14].

Outcome	No. of studies	No. of participants	Effect Estimate (95% CI)	Certainty of evidence (GRADE)	Comment	Typical guideline conclusion
All-cause mortality	15	5463	RR 0.88 (0.80–0.96)	⊕⊕⊕⊝ Moderate^a^	Hemodiafiltration likely reduces all-cause mortality	We suggest; or we suggest considering
Cardiovascular mortality	9	4761	RR 0.78 (0.66–0.92)	⊕⊕⊕⊕ High	Hemodiafiltration reduces cardiovascular mortality	We recommend; or we suggest

a Evidence could be downgraded once for indirectness (clinical heterogeneity in the treatment arms), or twice (if considering imprecision) or three times (if considering risk of bias).

The observation that mortality benefits are not paralleled by consistent reductions in hospitalization likely reflects the heterogeneous and health-system–dependent nature of hospitalization practices, rather than lack of treatment efficacy.

Recommendations arising from this body of evidence would therefore be framed along the lines that HDF (particularly high-volume HDF) likely reduces all-cause mortality, or may reduce all-cause mortality when considering greater uncertainty, and that HDF (online HDF in general) reduces, or likely reduces, cardiovascular mortality. On this basis, applying a GRADE-type framework, a guideline panel would likely issue a conditional recommendation (i.e. suggest) for the use of HDF to reduce all-cause mortality, and recommend or issue a conditional recommendation for HDF to reduce cardiovascular mortality. The final strength of recommendation would depend on contextual factors such as feasibility, resource requirements, equity, and patient preferences.

In summary, beyond CONVINCE, a first step is acknowledging appropriate formal assessments of the totality of evidence, while recognizing that uncertainty remains regarding indirectness (including generalizability and applicability across populations and settings), heterogeneity of treatment effects across patient subgroups, and contextual factors influencing implementation, and that additional large hard-endpoint trials may be limited by feasibility and ethical considerations.

### What questions remain unanswered? Research priorities beyond CONVINCE?

**Figure 1:**
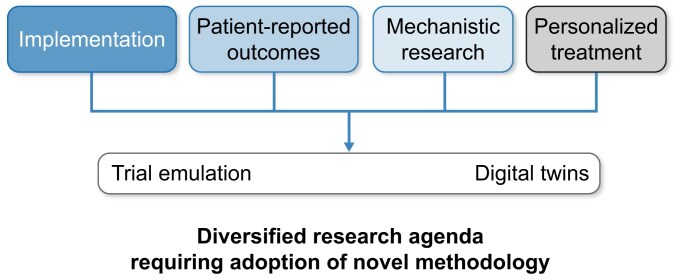
Research priorities after the CONVINCE trial and summaries of the totality of evidence on HDF. A conceptual framework outlining four key research priorities (implementation strategies, patient-reported outcomes, mechanistic understanding, and personalized treatment approaches). These pillars may all benefit from innovative methodological pathways, including trial emulation and digital twin simulations. These may support a diversified, modern and forward-thinking research agenda in dialysis.

A rational framework for post-CONVINCE research to address the remaining open questions includes in our view at least four broad priorities, outlined in Fig. 1.

These priorities are intended to support nuanced, patient-centered decision-making across renal replacement therapies, rather than to justify uniform adoption of a single modality.

### Priority number 1: implementation science

Implementation challenges for HDF are not unique to any single country and span multiple domains, as summarized in Table 3. In Europe, there is wide variation in the uptake of HDF reflecting infrastructure, reimbursement, and professional practice [22]. Countries such as the United States represent a particularly distinctive healthcare ecosystem, where HDF is not currently adopted.

**Table 3:** Global implementation barriers and strategies for adoption of hemodiafiltration.

Domain	Examples of barriers	Illustrative international context	Strategies to foster adoption
Structural	Limited HDF-capable machines; water infrastructure constraints	Variable infrastructure across countries	Phased roll-out; targeted capital investment
Regulatory	Absence of guideline endorsement; restrictive policies	Differences between national agencies (e.g. Europe versus US)	Guideline-based recommendations; regulatory alignment
Operational	Workflow complexity; staff workload concerns	Center-level variability (e.g. across Europe)	Protocol standardization; staff training
Financial/reimbursement	No differential reimbursement; misaligned incentives	Differential reimbursement (e.g. in Japan; e.g. bundled payments elsewhere)	Differential tariffs; pay-for-performance; value-based payment
Cultural/“people”	Clinician skepticism; lack of local champions	Marked differences in professional culture	Leadership engagement; clinician and nursing champions
Equity considerations	Rural or under-resourced centers	Regional disparities within and across countries	Equity-focused funding; regional support models

Overcoming country-level barriers, especially in settings with limited or no uptake of the technique, will require coordinated implementation strategies in which pragmatic research is a priority. This should then inform policy reform, education, and infrastructure investment.

Broadly, pragmatic implementation research embedded in routine dialysis care can identify the most relevant barriers to HDF adoption within specific health system contexts. These findings can inform targeted policy actions, including reimbursement reform, infrastructure investment, workforce training, and workflow redesign, grounded in real-world feasibility rather than theoretical assumptions. The alternative is that policy change may overtake research and be driven primarily by financial or operational considerations, leaving key research questions unanswered.

A foundational step in evaluating the current state of dialysis infrastructure is the assessment of system “readiness” and multi-stakeholder perspectives around HDF. This can be achieved through national surveys based on validated methods [e.g. those introduced by the Standardized Outcomes in Nephrology (SONG) initiative] [23, 24]. To move beyond descriptive assessment, Table 4 summarizes how key survey domains can be directly linked to actionable implementation strategies and policy levers to foster adoption of HDF.

**Table 4:** Using national readiness surveys to foster adoption of hemodiafiltration.

Readiness domain assessed	Examples of survey indicators	How findings can foster adoption	Implementation/policy levers informed
Infrastructure readiness	Availability of HDF-capable machines; water treatment capacity; space constraints	Identifies facilities ready for early adoption and those requiring phased upgrades	Targeted capital investment; phased roll-out strategies
Workforce and training	Staff familiarity with HDF; availability of trained nurses/technicians; perceived training burden	Highlights training gaps and informs development of standardized education programs	National training curricula; certification pathways; continuing education funding
Clinical workflow compatibility	Session duration constraints; scheduling flexibility; integration with existing protocols	Identifies workflow bottlenecks and feasible models of care	Workflow redesign; protocol standardization; staffing models
Organizational readiness	Leadership support; institutional priorities; perceived value of HDF	Identifies centers likely to act as early adopters or implementation hubs	Pilot-site selection; center-of-excellence models
Financial and reimbursement context	Perceived adequacy of reimbursement; cost concerns	Informs alignment of incentives with feasibility and outcomes	Reimbursement reform; pay-for-performance mechanisms
Equity and access considerations	Rural vs. urban capacity; underserved populations	Prevents widening disparities by identifying under-resourced settings	Equity-focused funding; regional support strategies

Multicenter pilot studies embedded in real-world settings will then be needed to test different HDF rollout strategies, including patient selection approaches, delivery logistics, staff training models, clinical workflows, and quality metrics. For countries where uptake of HDF is low, lessons from European centers where HDF is more broadly adopted can be used as a basis.

Health economics analyses will be essential. Country-specific research should evaluate how reimbursement schemes might be restructured to reward clinically appropriate use of HDF. Cost-effectiveness analyses at country level are needed, accounting for payer mix, hospitalization costs, and long-term outcomes, noting that current analyses are largely based on European data [25].

In the United States, policy modeling could include bundling HDF costs into the prospective payment system or introducing pay-for-performance metrics tied to interventions addressing cardiovascular survival and patient-centered outcomes, rather than surrogate endpoints of uncertain clinical relevance. In addition, HDF-related incentives within the Quality Incentive Program could serve as process-based measures linked to patient-relevant outcomes, warranting evaluation of their ability to complement existing quality metrics. Research is needed to determine whether such incentives could better align quality assessment with outcomes that matter most to patients, without undermining established measures.

Implementation research should also explore underserved populations, rural settings, and networks that may require tailored support (e.g. mobile water systems or telehealth integration) to achieve implementation of HDF. Studies on disparities in access to high-quality dialysis care are important to avoid exacerbating these conditions by inequitable HDF rollouts. Sustainability and equity are key considerations for HDF implementation. Transition from conventional HD may entail machine replacement, water system upgrades, and increased water and energy use, with associated environmental costs. In many low- and middle-income countries, the required capital investment may be prohibitive amid limited dialysis access and competing health priorities. These constraints highlight the need to evaluate HDF within a health system framework that balances clinical benefit with affordability, environmental impact, and opportunity cost, and to integrate cost-effectiveness, sustainability, and equity into future research and policy decisions. In summary, the capacity to implement strategically must be analyzed, country by country. This will require cross-sector collaboration, health systems research, regulatory engagement, and a strong focus on equity.

### Priority number 2: patient-reported outcomes (PROs): a fundamental frontier after convince

Despite dialysis being a life-sustaining intervention, survival alone does not fully reflect patients’ lived experience of dialysis treatment, and we have often failed to adequately address outcomes that patients prioritize most, such as quality of life, life participation, energy, mental health, and autonomy [27–30]. Initiatives such as SONG have helped demonstrate that trials and real-world studies incorporating PROs, which capture how patients feel and function, should be central to evaluating dialysis therapies. They are meaningful to patients and useful to clinicians for shared decision-making [31–33]. However, in both HD and HDF, PROs remain under-measured and inconsistently reported.

**Figure 2:**
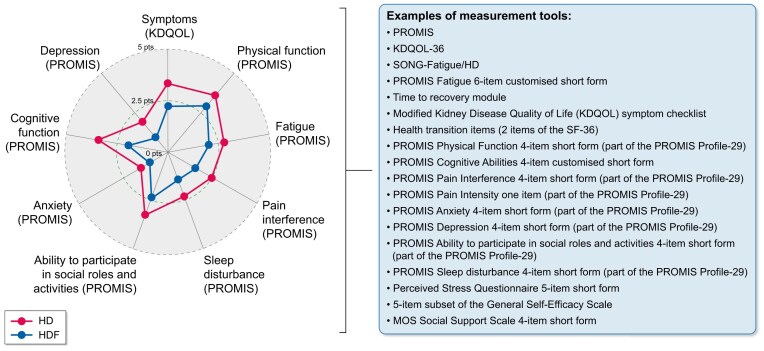
Patient-reported outcomes (PROs) in dialysis: potential impact of online hemodiafiltration (HDF). The radar plot shows key health domains, collected within the CONVINCE randomized trial, comparing change from baseline at 2 years between hemodialysis (HD; red) and online hemodiafiltration (HDF; blue). The domains (symptoms, physical function, fatigue, pain, sleep, social participation, anxiety, cognition, and depression) capture major aspects of patients’ daily experience. Each axis represents a different domain, and the distance from the center reflects the magnitude of change over time. Points plotted further away from the center indicate greater improvement (or more favorable outcomes), whereas points closer to the center indicate less improvement or worsening. The overall shape of each colored line therefore represents the profile of treatment effects across domains: a larger, more outward shape suggests broader or greater benefit across multiple aspects of health. Overall, the figure illustrates the potential for HDF to improve PROs across multiple important dimensions of health and well-being. Measurement tools relevant to these domains are listed, including PROMIS short forms, KDQOL-36, and SONG-Fatigue, which support routine and research-based assessment of PROs in dialysis care.

**Figure 3:**
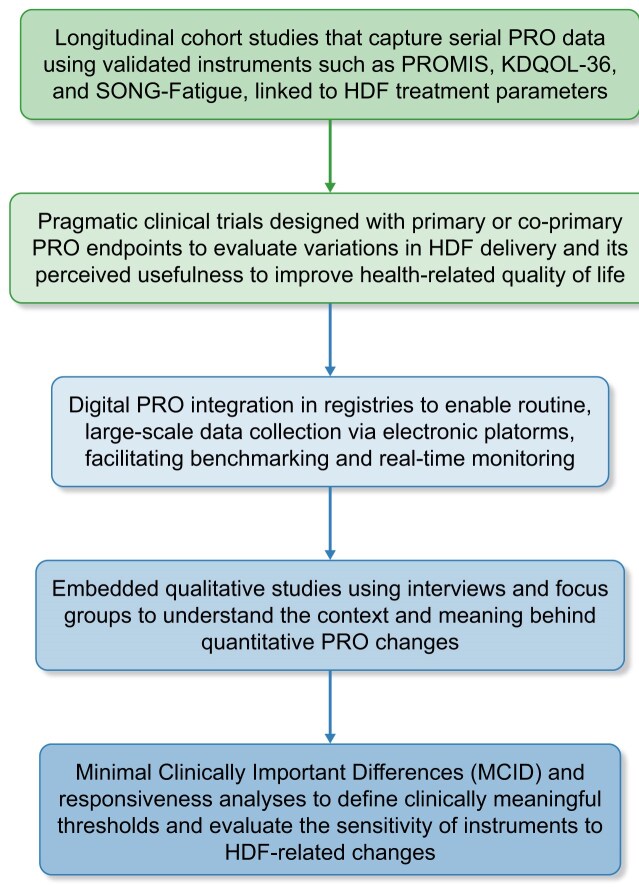
Key research opportunities to address patient-reported outcomes (PROs) in hemodiafiltration (HDF).

In comparison with earlier RCTs of HD (e.g. high versus low-flux HD, different dialysis regimens, etc.), the CONVINCE trial was the first in broadly incorporating PROs as measurable indicators of quality of life using validated instruments following qualitative interviews with patients and clinicians in full consideration of the International Consortium for Health Outcomes Measurements initiative [34]. The consideration of stakeholder interests, in an effort to close healthcare disparities and work toward a common framework to measure PROs in kidney care, was key in the proposal submitted to the EU for funding. Analysis included the PROMIS-29 v2.0 profile, encompassing key outcomes from the patients’ perspective [35, 36]. This comprised seven short forms, each with four items, measuring physical function, fatigue, sleep disturbance, depression, anxiety, pain interference, and the ability to participate in social roles and activities. Additionally, one item assessed pain intensity. Due to qualitative interviews with patients, two supplementary PROMIS items from the fatigue item bank were added to explore alternative ways to capture peridialytic symptoms related to fatigue and recovery. A customized version of the four-item PROMIS Cognitive Function Short Form was also included. CONVINCE showed that high-volume HDF slowed the decline in health-related quality of life compared to high-flux HD. The most notable benefit was in preserving perceived cognitive function, followed by physical function, reduction in pain intensity, and social participation [36]. These patient-reported benefits co-occurred with the observed survival advantage of HDF but the mechanisms remain unclear and no formal mediation analysis was performed. There is a need for longer follow-up and analysis of effects in real-world settings as well as the difficulty in measuring certain symptoms using currently available tools. With CONVINCE, the potential for what HDF could transform in the patients’ perspective appears large (Fig. 2) and warrants further investigation. A systematic research agenda for PROs in HDF, including six interlinked research approaches, is outlined in Fig. 3. This would require several study types and represents a complex but highly needed challenge and opportunity. To advance the role of PROs in HDF, we will need to design longitudinal cohort studies using standardized and validated instruments (e.g. computer-adaptive tests using PROMIS item banks, KDQOL-SF, SMaRRT-HD, SONG Fatigue, SONG Life Participation). These shall be backed by consistent survey development, supported by regular qualitative (re-)validation within a PRO framework. Pragmatic RCTs need to assess outcomes that are important to patients, including core symptoms such as fatigue, provided such trials don’t raise important ethical considerations (e.g. if they involve randomization to treatment strategies that may differ in survival benefit). Where equipoise is uncertain, alternative designs may be more appropriate. Important concepts that have not been explored systematically, such as dialysis recovery time, need to go through rigorous rounds of qualitative and quantitative testing before a suitable survey for use in RCTs and regular clinical assessment is recommended. Measurement of PRO endpoints will require integration of computer-adaptive PROs into routine care via digital platforms.

Observational and registry-based studies in centers using HDF offer an efficient way to assess PROs in real-world practice, capturing longitudinal changes across diverse populations. Integrating standardized PROs into dialysis registries enables large-scale, patient-centered comparisons with conventional hemodialysis.

An important barrier to the broader adoption of PROs internationally lies in the limited availability and cultural or linguistic validation of tools. Many translations remain unavailable or unvalidated, limiting their comparability across settings and populations. Efforts are also ongoing in this direction by the PROMIS international collaboration [37, 38].

It is clear that more efforts are needed to include PROs in research about HDF, which can help propel a shift in the field from viewing PROs as secondary endpoints to making them central in the evaluation of dialysis success [39]. This has already been advocated by patients, clinicians as well as multiple other stakeholders. Only when this is achieved will we be able to conclusively know the full value of HDF, not only from the relevant medical perspective of prolonging life, but also from the highly patient relevant perspective of improving the lives of those it serves.

### Priority number 3: mechanistic and translational studies: unlocking the biology of HDF

Although HDF has demonstrated advantages in several RCTs, the mechanistic pathways responsible for these benefits remain insufficiently elucidated [40]. Understanding these pathways is essential to ground observed effects in biological plausibility, guide innovation in membrane and machine design, and ultimately tailor therapy to biological phenotypes. Several mechanistic domains deserve expanded investigation, spanning solute clearance, inflammation, vascular biology, and patient-level physiological responses, as summarized in Table 5. HDF removes a broader spectrum of middle- and larger-molecule solutes, requiring studies to define which clearances translate into clinical benefit [41]. It may attenuate inflammatory and oxidative pathways, warranting deeper profiling of cytokine signatures and treatment-responsive phenotypes [42]. It also may improve endothelial biology through better shear stress and toxin removal, meriting evaluation of vascular and nitric oxide–related markers [43]. With HDF there may be enhanced intradialytic cardiovascular stability via improved plasma refill and ultrafiltration dynamics, needing mechanistic mapping across patient phenotypes [44]. HDF may also better modulate protein-bound uremic toxin (PBUT) pathways, requiring targeted metabolomics of PBUTs and precursor metabolites [45]. Neurocognitive function and cerebral perfusion may be supported by HDF with lower neurotoxic burden, necessitating studies using cerebral monitoring and cognitive testing [46]. HDF alters access flow and shear forces, prompting investigation of their effects on endothelial integrity and access durability [47]. Identifying mechanistically defined patient phenotypes may finally enable personalized HDF prescriptions and future precision-based innovation.

**Table 5:** Mechanistic domains of hemodiafiltration: biological rationale, research approaches, and translational clinical relevance.

Mechanistic domain/area of interest	Key biological hypothesis/rationale	Proposed methods/research needs	Putative clinical sequelae prevented or mitigated	Potential patient-relevant benefit
Solute removal profiles	HDF enhances clearance of middle- and large-molecular-weight uremic toxins beyond conventional HD, but the clinically relevant solute spectrum remains incompletely defined	Multi-omic profiling (proteomics, metabolomics); pilot clearance studies using mass spectrometry; linkage to clinical phenotyping	Chronic accumulation of uremic toxins contributing to cardiovascular injury, inflammation, fatigue, and long-term organ damage	Reduced global uremic burden; improved survival; attenuation of symptom burden (e.g. fatigue, malaise)
Systemic inflammation and endothelial health	HDF may reduce persistent microinflammation and preserve endothelial integrity through improved toxin and cytokine clearance	Inflammatory and endothelial biomarkers (CRP, IL-6, TNF-α, VCAM-1); endothelial function testing (e.g. flow-mediated dilation)	Inflammation-driven anemia and ESA resistance; accelerated atherosclerosis; vascular dysfunction	Improved ESA responsiveness; reduced cardiovascular events; improved energy levels and functional capacity
Oxidative stress	Enhanced removal of pro-oxidant molecules may reduce oxidative stress and downstream tissue injury	Redox biomarkers; oxidative stress panels; longitudinal profiling linked to outcomes	Oxidative myocardial and vascular injury; progression of cardiovascular disease	Improved cardiovascular stability; reduced long-term cardiac risk
Hemodynamic stability	HDF may improve plasma refill, ultrafiltration dynamics, and intradialytic hemodynamic tolerance	Continuous blood pressure monitoring; bioimpedance; cardiac biomarkers (troponin, NT-proBNP); perfusion studies	Intradialytic hypotension; myocardial stunning; repetitive end-organ ischemia	Improved treatment tolerance; reduced intradialytic symptoms (dizziness, cramps); protection of cardiac and cerebral function
Uremic toxin modulation (protein-bound uremic toxins)	HDF may better remove protein-bound uremic toxins inadequately cleared by HD	Targeted metabolomics; chromatographic techniques; kinetic modeling; symptom correlation studies	Neurotoxicity; pruritus; vascular and cardiovascular toxicity	Reduced pruritus; improved cognitive and neurological symptoms; potential cardiovascular benefit
Neurocognitive function and cerebral perfusion	Reduced neurotoxic exposure and improved intradialytic cerebral perfusion may confer neuroprotection	Functional MRI; near-infrared spectroscopy (NIRS); standardized neurocognitive testing	Accelerated cognitive decline associated with dialysis-related cerebral hypoperfusion	Preservation of cognitive performance; maintained independence and quality of life
Vascular access biology and shear stress	Altered flow dynamics and shear stress during HDF may influence endothelial signaling and access durability	Doppler ultrasound; shear stress biomarkers; computational flow modeling	Vascular access dysfunction, stenosis, thrombosis	Improved vascular access longevity; fewer access interventions and hospitalizations
Biological heterogeneity and treatment response	Patients differ in biological susceptibility and response to HDF-related mechanisms	Integrated biomarker profiling; subgroup analyses; phenotype-driven modeling	Non-response or disproportionate risk in specific patient subgroups	Basis for personalized HDF prescriptions; optimized benefit–risk balance

A nuanced understanding of all these mechanisms is especially important given that clinicians rely on biological plausibility when prescribing interventions. Fundamentally, this may well allow us to better tailor therapies. It is the job of translational research to bridge bedside observations with bench science. Ideally, mechanistic work would have to be embedded within future RCTs that need to be executed to further evaluate PROs as well as observational cohorts, to provide context and relevance. The long-term goal is to transform HDF from a one-size-fits-all modality into a biologically informed, precision kidney replacement therapy. Innovative methodologies, including use of digital twin technologies, may also be beneficial and are outlined in more detail in a subsequent section.

### Priority number 4: avoiding the missed opportunity of HD personalization with HDF

Personalization of dialysis care begins with appropriate modality selection across the full spectrum of renal replacement therapies, including conventional HD, HDF, alternative HD or HDF schedules, and home-based therapies, when supported by evidence and patient priorities.

Over the past two decades, several alternative HD strategies, including frequent, nocturnal, alternate-day, incremental, and home-based hemodialysis, have shown physiological plausibility and generated clinical and patient interest. However, evidence supporting these approaches has remained limited, with trials such as the Frequent Hemodialysis Network (FHN) studies being small, underpowered, and inconclusive [48, 49]. Overall, these strategies have been considered promising but unproven [50], and standard care has remained largely unchanged, centered on thrice-weekly in-center HD. This reflects broader challenges in evaluating personalized dialysis approaches, including logistical complexity, limited funding, and misalignment between trial design and patient priorities [51].

These limitations are exemplified by home hemodialysis, where strong patient interest contrasts with low uptake and a lack of robust randomized evidence [52–54], highlighting how structural, financial, and cultural barriers can hinder the evaluation and implementation of personalized strategies.

In this context, the emergence of HDF presents an opportunity to advance personalization in dialysis care; however, without deliberate efforts, there is a risk of repeating similar limitations in evidence generation and implementation.

With the superiority of HDF now established, the question is: will we just aim for a standard thrice-weekly HDF regimen delivered in-center for at least 4 h per session? Or will we also be able to test more personalized regimens, whether it be frequent, nocturnal, alternate-day, incremental, and/or home-based delivery of HDF, or more? Hemodiafiltration presents several possibilities for personalization, including adjusting convective volume. Yet, unless explicitly studied, these opportunities will be lost to the same inertia.

Several key questions remain regarding personalization of hemodiafiltration. These include whether benefits vary with convective dose, whether a dose–response or plateau effect exists, and whether lower convective volumes may provide meaningful benefits beyond mortality in selected patients. It also remains unclear to what extent observed benefits are modified by vascular access quality, particularly given lower catheter use in CONVINCE than in many real-world settings.

Additional uncertainties include how HDF efficacy should be monitored beyond Kt/V in routine practice, the optimal use of pre- versus post-dilution HDF across different clinical and geographic contexts, and whether patient characteristics such as age, comorbidity burden, dialysis vintage, vascular access, or inflammatory status should guide prioritization of HDF initiation. Addressing these questions may enable more targeted, efficient, and equitable adoption.

New trials, especially with more modern designs, could be used to address this. Their specific characteristics are shown in Table 6. However, even doing so we would most probably not succeed at doing enough and well-powered RCTs, as was the case with HD. The fundamental challenges facing the research enterprise [51] remain and patients, caregivers, and clinicians perspectives and priorities are often not aligned [53, 54].

**Table 6:** Trial designs that could be used to address evaluation of benefits and harms of personalized hemodiafiltration (HDF) treatment compared to standard HDF.

Methodological approach	Description
Adaptive trial designs for treatment schedules	Trials that allow modifications (e.g. dose, schedule) during the study based on interim results, optimizing treatment regimens in real-time.
N-of-1 trials	Single-patient crossover trials where an individual undergoes multiple treatment periods, allowing personalized assessment of efficacy.
Cluster-randomized pragmatic trials	Trials randomizing groups (e.g. dialysis centers) rather than individuals, enabling real-world comparisons across systems with minimal disruption, restricted to research questions where genuine uncertainty remains (e.g. implementation strategies, dosing, or patient subgroups), and where comparisons do not require withholding HDF in settings where it is considered beneficial.
Registry-based personalization studies	Observational or quasi-experimental studies using registry data to tailor treatments to subgroups based on real-world effectiveness.
Patient-informed trial design and co-design of trials with patients	Designs that integrate patient preferences and priorities into study protocols, improving relevance and engagement, co-designed with support of consumer advisory boards.
Hybrid effectiveness-implementation trials	Studies that test both clinical effectiveness and strategies for implementation, bridging research and practice to accelerate adoption.

### Examples of innovative methods to advance future research around the mechanisms, the outcomes and the personalization opportunities of treatment with HDF

We believe embracement of novel research methods is probably key to real advancement in this area of research. Without deliberate modern approaches that go beyond the constraints of traditional RCTs or basic science models, we risk repeating a cycle of inertia like the one that happened with HD: underpowered trials, delayed translation, and unrealized patient benefit.

*Target trial emulations (applicable to research priorities 1 and 2):* an increasingly recognized approach when new RCTs are difficult to conduct or their applicability in real-world settings is limited is target trial emulation using real-world data [55, 56]. Leveraging large-scale dialysis databases, researchers can emulate hypothetical randomized trials by applying rigorous protocols for eligibility, follow-up, treatment allocation, and confounder adjustment. This can approximate the estimation of causal effects of different dialysis regimens (e.g. HD versus HDF, extended versus standard sessions) without the need for de novo trial recruitment. Patient data are derived from real-world settings, increasing external validity.

However, these approaches rely on the availability of sufficiently large and representative datasets in which both interventions of interest are routinely used. Because target trial emulation involves the application of strict eligibility criteria and adjustment procedures, substantial reductions in effective sample size may occur. As a result, their feasibility depends on widespread adoption and adequate representation of the treatments being compared. In the context of HDF, these may be available in European settings, where the technique is more broadly adopted, while this may imply that broader implementation, potentially including heterogeneous or non-systematic uptake, may be required before such methods can robustly address more granular clinical or policy-relevant questions in countries like the United States.

Complementing this, synthetic control arms can serve as comparators when traditional two-arm trials are not feasible. For instance, a single-arm study evaluating nocturnal HDF could use registry-based matched cohorts as external controls. Similarly, agent-based simulation models, which are computational frameworks where individual “agents” (e.g. patients, dialysis units, clinicians, or payers) behave according to defined rules and interact with their environment, can project the impact of scaling HDF across highly diverse populations and healthcare systems, accounting for confounders such as age, comorbidity, dialysis vintage, and system-level constraints including staffing and infrastructure. These models offer a practical way to assess expected uptake, cost-effectiveness, and unintended consequences of different implementation strategies.

Co-designed simulation scenarios with input from patients, clinicians, payers, and policymakers will be necessary to ensure that models reflect real-world barriers, including transportation, socioeconomic disparities, and patient burden. Integrating behavioral and preference data (e.g. dropout risks, treatment fatigue) could help move beyond idealized efficacy to more realistic estimates of effectiveness with HDF (and also with HD).

*Digital twins and virtual trials for HDF research (applicable to priorities 2, 3, and 4)*: digital twins have gained attention across the life sciences. Their relevance in HDF may be specific to several unresolved challenges that are difficult to address using conventional designs, around personalization in settings where large, long-term randomized trials are increasingly impractical. In this context, digital twins refer to computational representations of dialysis patients that integrate clinical, biological, and treatment data to simulate responses to HDF-specific prescription parameters.

The technology of digital twins has already been used for mechanistic studies, although not specifically in the renal replacement setting [57, 58]. This remains as we argued earlier a fundamental priority for HDF research perceived by nephrologists. They have not yet been used for RCTs, but simulation cohorts were generated to compare interventions, simulating trial arms *in silico*, replicating trial effect sizes across different populations with their digital twins [59, 60]. Given the fast pace at which AI innovation is progressing, it may be hypothesized that this field of research will rapidly advance in some form and we may randomize digital twins of patients rather than real patients in future RCTs.

**Figure 4:**
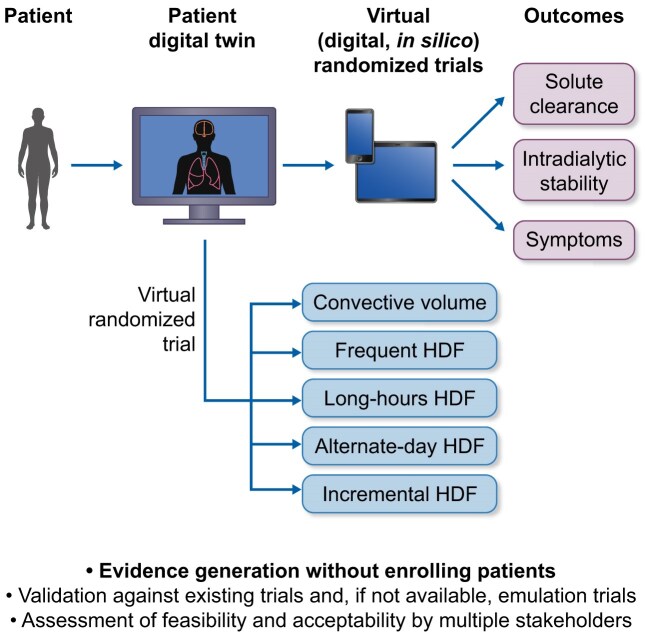
Using digital twins to simulate personalized hemodiafiltration (HDF) trials. This conceptual flowchart illustrates how digital twin technology can support virtual clinical trials for personalized HDF treatment. The process begins with the integration of multimodal patient data, including clinical characteristics, biomarkers, and dialysis parameters, into computational models that create individualized digital replicas. These digital twins are then used to simulate various HDF regimens (e.g. convection volume, session frequency, treatment duration) and predict outcomes such as solute clearance, intradialytic hemodynamic stability, cognitive function, and patient-reported outcomes. By comparing simulated responses across treatment strategies and patient subgroups, this approach enables rapid, cost-efficient, and ethically sound evaluation of personalized HDF interventions—without exposing real patients to experimental risk. The model allows for iterative refinement, predictive validation, and decision support, accelerating the translation of personalized dialysis into clinical practice.

A highly transformative work hypothesis using digital twins in the setting of HDF personalization is presented in Fig. 4. Here, the focus is on simulating HDF-specific treatment parameters and their impact on outcomes including solute clearance, hemodynamic stability, fatigue, and PROs. It could be possible to model patient subtypes, such as those with high inflammation, poor vascular access, or low residual kidney function, and predict differential responses to personalized HDF strategies.

Tables 7 and 8 summarize a proposed HDF-focused framework for developing and operationalizing digital twins for virtual treatment evaluation, including feasibility, ethical considerations, and stakeholder acceptability.

**Table 7:** Framework for developing digital twins of dialysis patients for virtual treatment evaluation.

Stage/component	Description	Examples/technologies	Purpose/outcome
Initial simulation setup	Digital twins (simulated dialysis patients) are created and assigned to different HDF protocols	Alternate-day HDF, nocturnal HDF	Enables virtual comparison of treatment effects without real-patient randomization
Modeling framework for digital twins	AI architectures simulate patient trajectories over time	RNNs, transformers, TARNet, DragonNet	Predict cardiovascular outcomes, symptom burden, survival, treatment response
Data sources for model training	Multi-modal clinical and physiological data to feed simulation models	EHRs, lab values, longitudinal dialysis data, sensor data	Creates a dynamic, realistic representation of patient physiology and outcomes
Computational platform requirements	Secure, scalable infrastructure for data ingestion and modeling	GDPR-compliant cloud-based system	Ensures privacy, enables large-scale real-world dataset integration
Data volume and origin	Large datasets from dialysis networks across diverse regions^a^	Thousands of patients, real-world clinical repositories	Supports robust model training and generalizability of digital twins
Synthetic data generation	Creation of artificial datasets to enhance model development	PATE-GAN, BGMM-OCE, TVAE	Produces high-quality synthetic samples for training and validation
Hybrid generation strategies	Combining synthetic-data models for improved realism and coverage	Probabilistic + generative model architectures	Increases diversity and fidelity of simulated patient populations
Fairness optimization	Bias mitigation for more ethical digital twin modeling	Post-hoc fairness adjustment techniques	Promotes equitable outcomes across underrepresented groups

^a^Hemodialysis is a well-recognized “data-rich” environment.

**Table 8:** The four phases of our proposed methodological framework for virtual clinical trials in hemodialysis/hemodiafiltration.

Phase	Purpose	Examples of target intervention	Data sources	AI modeling techniques	Outcomes to be assessed	Validation strategy
I	Simulate virtual RCTs of personalized dialysis strategies	Alternate-day, nocturnal, frequent, and incremental HDF versus standard hemodialysis	Database from a renal care provider (public or private)	Deep learning architectures: RNNs, Transformers. Causal inference frameworks: TARNet, CFRNet. Synthetic data augmentation: PATE-GAN, BGMM-OCE, TVAE (and hybrid versions). Fairness adjustments to reduce bias (e.g. at data level using synthetic data at AI model level using fairness re-weighting).	All-cause and cardiovascular mortality- Hospitalizations, QoL, adherence	Comparison with historical RCTs (FHN, CONVINCE)-metrics to be calculated for treatment effects include the ATE, ITE, RMSE
II	Augment underpowered ongoing RCTs with digital twin ‘virtual arms’	Middle cut-off HDx (complementing the MOTheR* trial)	MOTheR* RCT (700 real patients) + 1114 digital twins	Bayesian hierarchical modeling, Propensity score methods, GNNs for treatment effect robustness	Mortality, MACE, hospitalization, dialysis efficiency	Concordance between virtual and real outcomes, Sensitivity analyses across subgroups
III	Conduct full-scale virtual Phase III trials using Phase I/II data only	HIF stabilizers for anemia versus standard ESA treatment	Phase I/II data + real-world registry and EHR data	DragonNet, Monte Carlo simulations, Counterfactual modeling	Survival, stroke, MI, thrombosis, QoL	Comparison with existing Phase III trials-Bias detection and uncertainty estimation
IV	Assess feasibility, acceptability, and ethical perceptions around using virtual trials	-	Interviews with clinicians, patients, payers, regulators (SONG-based protocols)	Survey/thematic analysis	Barriers and facilitators to adoption, trust and regulatory alignment	Qualitative coding (HyperRESEARCH, triangulation with model outputs)

*The MOTheR HDx study is an open-label, multicenter, prospective, 1:1 randomized, parallel-group non-inferiority interventional trial (*n* = 700) comparing expanded hemodialysis (HDx, middle cut-off dialyzer) with online hemodiafiltration (OL-HDF).

Counterfactual simulations could be used to estimate how different dialysis strategies might affect patient outcomes under various scenarios. Outcomes of interest would include all-cause and cardiovascular mortality, major adverse cardiovascular events, hospitalizations, treatment adherence, and PROs. The effects of different treatment approaches could then be compared across simulated patient populations.

To ensure credibility, these simulation results would need to be compared against findings from completed randomized trials, such as the FHN [48] and CONVINCE [10] studies. Additional analyses would be performed to test the stability and reliability of the results under different assumptions.

Importantly, the acceptability and feasibility of these approaches should also be evaluated by engaging patients, clinicians, regulators, and payers. This could be achieved through structured interviews and qualitative research methods, to understand whether such approaches are considered trustworthy, useful, and aligned with clinical and regulatory expectations.

We emphasize that at present digital twins are not proposed as replacements for randomized trials, but as hypothesis-generating and prioritization tools to support HDF-specific research when traditional designs face ethical, logistical, or feasibility constraints.

## CONCLUSION

HDF is at a crucial clinical and scientific turn-point beyond CONVINCE. Whether we realize its full potential will depend upon our ability to innovate how we study, fund, and deliver dialysis care. Replacement of existing renal replacement therapies will not happen “tout court,” but evidence-based guideline development and prioritization of future research in areas where uncertainty remains are fundamental. The future lies way beyond CONVINCE. It requires creativity and commitment to evidence-informed progress that our medical and scientific community can, knows how to and must fully exercise, “outside of the box,” because patients deserve it and timing is appropriate.

## Data Availability

The data underlying this article will be shared on reasonable request to the corresponding author.
